# Supplementary material for: Unveiling Hidden Risks: Intentional Molecular Screening for Sexually Transmitted Infections and Vaginosis Pathogens in Patients Who Have Been Exclusively Tested for Human Papillomavirus Genotyping
Source: Microorganisms. 2023 Oct 30;11(11):2661. doi: 10.3390/microorganisms11112661 (PMC10672824; doi:10.3390/microorganisms11112661)
Supplement: Supplementary file 1 [file microorganisms-11-02661-s001.zip › microorganisms-2625411-supplementary materilas.pdf]

Unveiling Hidden Risks: Intentional Molecular Screening of STI and Vaginosis Pathogens in patients who were exclusively tested for HPV Genotyping test

Supplementary Table S1. The primer sequences for COMPLEMENTARY STI PANEL.

Complementary Table 1. Primers sequences designed for multiplex PCR assay**.**

Pathogen	Primer Name	Sequence
Molluscum Contagiosum Virus	M1_MCV_F1	GCGCGTTTTCGCGGCCTTAAAAT
	M1_MCV_R1	ATAATCGTCACGCGCACGTCGG
Herpes simplex virus	M1_HSV_F1	GCCCTGGTCGACCTGCTGTTTT
	M1_HSV_R1	GTCTGCTCAGTTCGGCGGTGAG
Treponema pallidum	Primer KO	CAGAGCCATCAGCCCTTTTCA
	Primer KO4	GTCTGCTCAGTTCGGCGGTGAG
Haemophilus spp.	M1_Hadu_F1	AGGCTGACCAAGCCGACGATCT
	M1_Hadu_R1	AGGCCTTCTTCATTCACGCGGC
Staphylococcus aureus	M1_Stau_F1	TTCTGGTGCTGGATCTCGACCT
	M1_Stau_R1	CGTGCAATTGCTGACCAAGCACG
Klebsiella spp.	M1_Klgr_F1	GGTTCGAAAGCGGAAGCCTGGG
	M1_Klgr_R1	TGATCGGGGTCATCTTGCGGGT
